# Pediatric Primitive Neuroectodermal Tumors of the Central Nervous System Differentially Express Granzyme Inhibitors

**DOI:** 10.1371/journal.pone.0151465

**Published:** 2016-03-10

**Authors:** Jeroen F. Vermeulen, Wim van Hecke, Wim G. M. Spliet, José Villacorta Hidalgo, Paul Fisch, Roel Broekhuizen, Niels Bovenschen

**Affiliations:** 1 Department of Pathology, University Medical Center Utrecht, 3584CX, Utrecht, The Netherlands; 2 Laboratory of Translational Immunology, University Medical Center Utrecht, 3584CX, Utrecht, The Netherlands; 3 Institute of Pathology, University Medical Center Freiburg, 79106, Freiburg, Germany; University of Navarra, SPAIN

## Abstract

**Background:**

Central nervous system (CNS) primitive neuroectodermal tumors (PNETs) are malignant primary brain tumors that occur in young infants. Using current standard therapy, up to 80% of the children still dies from recurrent disease. Cellular immunotherapy might be key to improve overall survival. To achieve efficient killing of tumor cells, however, immunotherapy has to overcome cancer-associated strategies to evade the cytotoxic immune response. Whether CNS-PNETs can evade the immune response remains unknown.

**Methods:**

We examined by immunohistochemistry the immune response and immune evasion strategies in pediatric CNS-PNETs.

**Results:**

Here, we show that CD4^+^, CD8^+^, γδ-T-cells, and Tregs can infiltrate pediatric CNS-PNETs, although the activation status of cytotoxic cells is variable. Pediatric CNS-PNETs evade immune recognition by downregulating cell surface MHC-I and CD1d expression. Intriguingly, expression of SERPINB9, SERPINB1, and SERPINB4 is acquired during tumorigenesis in 29%, 29%, and 57% of the tumors, respectively.

**Conclusion:**

We show for the first time that brain tumors express direct granzyme inhibitors (serpins) as a potential mechanism to overcome cellular cytotoxicity, which may have consequences for cellular immunotherapy.

## Introduction

Embryonal tumors of the central nervous system (CNS), i.e. medulloblastoma, atypical teratoid rhabdoid tumor, and CNS primitive neuroectodermal tumors (PNET), are the most common malignant primary brain cancers in children and account for approximately 20% of all pediatric brain tumors [1]. Histologically they appear as small round blue progenitor cell tumors, but biologically and molecularly they are distinct entities [2, 3]. CNS-PNETs have an annual incidence of 0.62 per 1,000,000 children in the USA [4]. They are treated like high-risk medulloblastomas, resulting in a 5-year disease free survival of 15–50%, which is worse than medulloblastomas (5-year disease free survival of ~80%) [5–7]. In analogy to other brain tumors, like gliomas, immunotherapy might be key to improve survival in CNS-PNETs. Therefore, it is of importance to understand the immune response against CNS-PNETs. Efficient killing of CNS-PNETs during immunotherapeutic protocols can only be achieved when potential tumor-associated mechanisms to evade recognition or killing by the immune system are overcome. It has been well established that cancers employ multiple mechanisms to evade our immune system, making them less susceptible for immunotherapy [8]. Evidence for the existence of immune evasion strategies in brain tumors comes from gliomas and medulloblastomas, showing that subtypes downregulate MHC-I expression pointing to evasion from T cell-mediated anti-tumor immunity [9, 10] or lack CD1d expression to evade NKT cell recognition [11]. Moreover, expression of intracellular apoptosis inhibitors (e.g. caspase inhibitors) to escape from death receptor-induced apoptosis and granzyme-mediated killing pathways [8] predicts a worse clinical outcome and poor response to cellular immunotherapy [12, 13]. Whether CNS-PNETs can evade the immune response remains to be elucidated. The aim of this study is to survey several cases of pediatric CNS-PNET for tumor-infiltrating lymphocytes and immune evasion molecules, allowing to facilitate prediction of the tumor response to immunotherapy.

## Materials and Methods

### Patients

We examined by immunohistochemistry the cytotoxic immune response and immune evasion strategies in seven primary pediatric CNS-PNETs operated between 1998–2014 at the University Medical Center Utrecht (Utrecht, The Netherlands). Patient characteristics are shown in Table 1. The study material was derived from the archive of the Department of Pathology of the University Medical Center Utrecht, Utrecht, The Netherlands and distributed by the Biobank of the Department of Pathology. The biobank is overseen by the institutional medical ethical review board.

**Table 1:** Patient characteristics.

Case	Gender	Age (years)	Location	Histology^†^	Survival (months)	GFAP*	NeuN*	Synaptophysin*	Ki-67*	Ini1*	β-catenin
**1**	Female	2	Frontal lobe bilateral	CNS PNET, NOS	Died (21)	0	0	100	75	100	Cytoplasmic
**2**	Female	2	Insula left	CNS PNET, NOS	Died (2)	<1	0	90	50	100	Cytoplasmic
**3**	Female	2	Frontal-temporal lobe right	CNS PNET, NOS	Died (10)	0	0	<1	95	100	Cytoplasmic
**4**	Male	9	Frontal lobe right	CNS PNET, NOS	Died (5)	<1	0	90	65	100	Cytoplasmic
**5**	Female	17	Frontal lobe/ regio pinealis	CNS PNET, pineoblastoma	Alive (50)	0	0	100	10	100	Cytoplasmic
**6**	Female	7	Parieto-ocipital lobe right	CNS PNET, Ependymoblastoma	Died (25)	40	5	30	5	100	Cytoplasmic
**7**	Female	2	Insula left	CNS PNET, Ependymoblastoma	Died (4)	75	<1	0	75	100	Cytoplasmic

^†^Tumors were reclassified according to the 4^th^ edition of the WHO classification of tumors of the central nervous system.

*Values are displayed as percentage positive tumor cells.

Since we are using archival pathology material which does not interfere with patient care and does not involve physical involvement of the patient, no ethical approval is required according to Dutch legislation [14]. Use and storage of anonymous or coded left over material for scientific purposes is part of the standard treatment contract with patients and therefore informed consent procedure was not required according to our institutional medical ethical review board, this has also been described by van Diest [15].

### Immunohistochemistry

Immunohistochemistry was carried out on 4μm thick formalin fixed paraffin embedded consecutive sections. For tumor classification, all stainings (GFAP, Synaptophysin, Neu-N, Ini1, β-catenin, Ki-67) were repeated using an automated immunostainer (Benchmark Ultra, Ventana, Roche). All other stainings were performed manually, except CD4 and SerpinB1 that were stained using the immunostainer. After deparaffination and rehydration, endogenous peroxidase activity was blocked for 15 min in a buffer solution pH5.8 containing 0.3% hydrogen peroxide. After antigen retrieval, i.e. boiling for 20 min in 10 mM citrate pH6.0, or Tris/EDTA pH9.0, a cooling off period of 30 min preceded the primary antibody incubation. All primary antibodies were diluted in PBS containing 2% BSA and incubated for 1h at room temperature (NKp46 and SERPINB4 were incubated o/n at 4°C). Antibody dilutions can be found in Table 2. The signal was amplified using Brightvision poly-HRP anti-mouse, rabbit, rat (DPVO-HRP, Immunologic) or in case of NKp46 with rabbit anti-goat HRP (DAKO), or in case of SERPINB4 the Novolink kit (Leica), and developed with diaminobenzidine followed by counterstaining with haematoxylin, dehydration in alcohol and mounting. The γδ-TCR staining was performed by boiling in 10 mM citrate pH6.0 for 2 min in a pressure cooker, followed by washing with TBS-T for 5 min (DAKO) and blocking with 5% Human serum in PBS for 30 min and avidin-biotin blocking (DAKO) for 20 min prior to antibody incubation. Signal amplification was performed using the REAL Detection System, Alkaline Phosphatase/RED, Rabbit/mouse (DAKO) followed by counterstaining with haematoxylin and mounting. Appropriate positive and negative controls were included in all stainings.

**Table 2:** Overview of used antibodies.

	Antigen retrieval	clone	Company	dilution
GFAP	EDTA	27G12	Novocastra	1:200
Synaptophysin	EDTA	6F2	Dako	1:1,000
Neu-N	EDTA	Mab377	Chemicon	1:500
Ini1	EDTA	CL25/Baf47	BD Biosciences	1:100
β-catenin	EDTA	17C2	Novocastra	1:20
Ki-67	EDTA	M7240	Dako	1:100
HLA-A	Citrate	HCA2	Ref. [29]	1:100
HLA-B	EDTA	HC10	Ref. [29]	1:200
CD1d	EDTA	NOR3.2	Thermo Scientific	1:100
β2-microglobulin	None	A072	Dako	1:600
CD3	EDTA	A452	Dako	1:100
CD4	EDTA	SP35	CellMarque	1:50
CD8	Citrate	M7103	Dako	1:50
CD20	Citrate	M0755	Dako	1:800
FOXP3	EDTA	150D/E4	Ebioscience	1:100
NKp46	EDTA	AF1850	R&D Systems	1:200 o/n
γTCR	Citrate	Gamma3.20	Thermo Scientific	1:40
CD34	Citrate	QBEnd10	Immunotech	1:800
Bcl-2	Citrate	M0887	Dako	1:200
Survivin	Citrate	71G4B7	Cell Signaling	1:400
SERPINB1	Citrate	Ab47731	Abcam	1:800
SERPINB4	EDTA	10C12	Santa Cruz	1:50 o/n
SERPINB9	Citrate	Clone 17	Ref. [30]	1:400
Granzyme B	Citrate	GB7	Ref. [31]	1:250

### Scoring of immunohistochemistry

Reclassification of cases was performed independently by two experienced neuropathologists (WGSS and WVH). All scoring was done blinded to patient characteristics and results of other staining by three independent observers (JFV, WGSS, WVH). Analysis of the immune influx was performed on whole slides at 20x magnification. Immune influx was corrected for the size of tissue on the slide and the tumor percentage as calculated with the manufacturers algorithm based on digitalized immunochemical slides using a digital slide scanner (Aperio Technologies Inc.). Serpin expression was scored as present (+) or absent (-), because serpins were uniformly expressed throughout the tumor.

### Statistics

Statistical analysis was performed using IBM SPSS version 21 (SPSS Inc.). Descriptive statistics were examined as median and inter quartile range (IQR) taking all patients into account. No statistical tests or survival analyses were performed due to the number of patients included in this study.

## Results and Discussion

**Fig 1:**
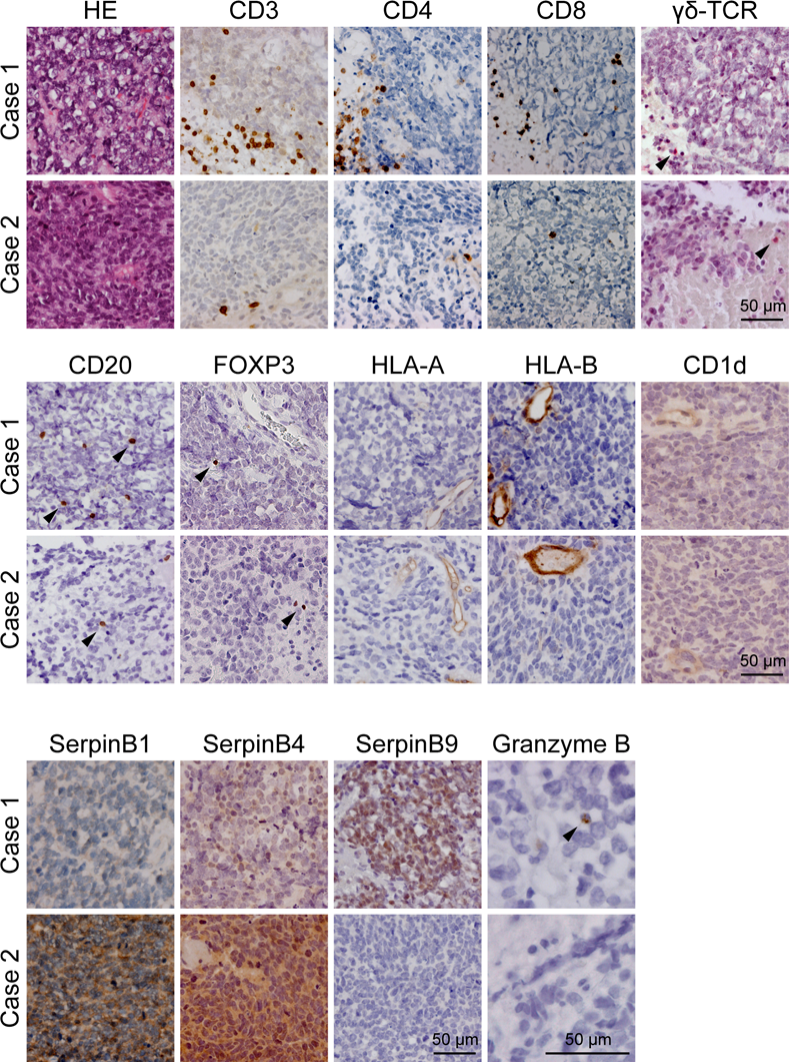
Immune (evasion) markers in cases of CNS-PNET. Immunohistochemical staining of immune markers in two CNS-PNET cases. Note that SerpinB9 expression coincides with high numbers of activated GrB ^+^ CTLs in case 1 versus low numbers of activated GrB ^+^ CTLs in case 2. Size bar equals 50μm.

We found a substantial and highly variable influx of immune cells from the (tumor-associated) vasculature into the tumor tissue, i.e. 28.5 (43.7) [median (IQR)] CD3^+^ T-cells and 6.4 (13.3) CD20^+^ B-cells per 2mm^2^ tumor tissue. Normal brain parenchyma did not contain CD3^+^ T-cells or CD20^+^ B-cells. Data on individual patients is shown in Table 3. The CD3/CD20 ratio in the tumor tissue was 7.0 (12.6). The immune cell influx was independent of the vascular density. Leucocytosis or leucocytopenia prior to surgery, as determined by routine laboratory blood tests, was absent in all patients. The CD3^+^ T-cell population consisted of 9.6 (27.8) CD8^+^ T-cells and 8.7 (16.1) CD4^+^ T-cells per 2mm^2^ tumor tissue [CD8^+^ /CD4^+^ T-cell ratio of 1.2 (0.3)]. Next, we studied the influx of γδ-T-cells, an additional cytotoxic lymphocyte subset [16, 17]. We detected low numbers of γδ-T-cells in CNS-PNET [0.1 (0.2)]. Recruitment of NKp46^+^ NK-cells could not be detected in CNS-PNET, which is in line with results in other CNS tumors [18, 19]. The activity of cytotoxic tumor-infiltrating lymphocytes was examined by granzyme B (GrB) expression, resulting in a GrB-positive CTL fraction of 8.4% (8.4%). One patient (Case 1, Fig 1) had 29.0% GrB positive CTLs whereas all the other patients had <10% GrB positive CTLs (Case 2 is shown as an example, Fig 1).

**Table 3:** Results on infiltrating immune cells and Granzyme inhibitor expression of the individual patients.

Case	CD3^+^	CD4^+^	CD8^+^	CD20^+^	FOXP3^+^	NKp46^+^	γTCR^+^	CD3/CD20 ratio	CD8/CD4 ratio	GrB^+^ CTL fraction	SERPINB1	SERPINB4	SERPINB9
**1**	15.92	5.41	6.37	6.39	0.33	0	0.74	2.49	1.18	0.29	-	-	+
**2**	11.14	4.55	3.37	1.11	0.32	0	0.31	10.00	0.74	0.066	+	+	-
**3**	6.41	2.55	3.59	10.81	0.061	0	0.061	0.59	1.41	0.014	-	+	-
**4**	110.03	44.22	51.06	15.78	0.14	0	0.24	6.97	1.15	0.099	+	+	-
**5**	54.86	20.63	31.40	3.95	1.30	0	n.a.	13.88	1.52	0.084	-	-	+
**6**	38.83	15.27	16.27	2.51	0.23	0	0.028	15.45	1.07	0.015	-	-	-
**7**	28.46	8.70	9.57	21.85	0.17	0	0.26	1.30	1.10	0.099	-	+	-

Values are displayed as number of immune cells per 2mm^2^ tumor tissue.

+ and - indicates presence or absence of expression of SERPINs, respectively

Given the modest cytotoxic lymphocyte activation, we wondered whether CNS-PNETs downregulate MHC-I (HLA-A and HLA-B) and/or CD1d to avoid CTL, NKT, and/or CD1d-restricted γδ-T-cell [20] recognition. Whereas normal brain tissue, blood vessels, and infiltrating immune cells readily express these HLA molecules and CD1d, we found that HLA-A, HLA-B, and CD1d expression was not detectable in all CNS-PNET cases (Fig 1). In addition, β2-microglobulin expression was decreased concordantly (data not shown). We detected low numbers of FOXP3^+^ regulatory T-cells [0.2 (0.3) per 2mm^2^ tumor tissue], which may contribute to attenuation of immune activity. Together, these data suggest that pediatric CNS-PNETs may evade recognition by cytotoxic immune cells.

Granzymes are the major tumor killing molecules secreted by cytotoxic cells. In humans, five granzymes (i.e. GrA, GrB, GrH, GrK, and GrM) exist with distinct substrate specificities and only partially overlapping routes of apoptosis induction [21]. Certain tumors can express serine protease inhibitors (serpins) to directly block granzyme activity. It has been well established that SERPINB9 (GrB inhibitor also called PI-9) expression in melanomas correlates with worse clinical outcome and poor response to immunotherapy [13]. For brain tumors in general, it remains unknown whether granzyme inhibitors are expressed. Intriguingly, we now show that 29% of CNS-PNETs had expression of the GrB inhibitor SERPINB9 (Fig 1). We found that SERPINB9 expression was absent in the normal brain parenchyma, indicating that SERPINB9 expression is acquired during tumorigenesis. Remarkably, high SERPINB9 expression was constrained to the case with high cytotoxic lymphocyte activation. We also addressed the expression of the GrH and GrM inhibitors (SERPINB1 [22] and SERPINB4 [23], respectively) in CNS-PNETs. Like SERPINB9, expression of SERPINB1 and SERPINB4 was acquired in CNS-PNETs. We found SERPINB4 expression in four CNS-PNETs, of which two cases turned out to be also SERPINB1 positive (Fig 1, Table 1). These data suggest that SERPINB1, SERPINB4, and SERPINB9 are differentially expressed in pediatric CNS-PNETs. Interestingly, besides these serpins, all tumors had high expression of Survivin (caspase-3 inhibitor) and Bcl-2 (cytochrome c release inhibitor), which are both downstream inhibitors of granzyme-induced apoptotic pathways.

The molecular mechanisms and physiological relevance of intracellular serpin expression in pediatric brain tumors remains unknown. SERPINB9, B4, and B1 are known to inhibit granzyme B, M, and H, respectively, and are therefore likely to contribute to evasion of granzyme-induced cytotoxicity [22–24]. However, we cannot exclude that these serpins also play other roles in tumorigenesis. Recently, Valiente et al showed that expression of neuroserpin and SERPINB2 are essential for developing brain metastases in patients with lung and breast cancer. Expression of these serpins inhibited plasminogen activator and FAS-L induced cytotoxic cell death by reactive astrocytes and is therefore a marker for poor prognosis [25]. Furthermore, SERPINB1 expression in glioma and glioblastoma cell lines seems to be a marker for good prognosis, because it inhibits tumor cell migration and invasion [26]. Finally, SERPINB4 expression in patients with squamous cell carcinomas is associated poor prognosis and SERPINB4 can inhibit both radiation- and TNF-induced apoptosis, probably by inhibition of the p38 MAPK pathway and the proteolytic activity of endogenous cathepsin G, respectively [27, 28]. Whether CNS-PNETs with high serpin expression are more prone to be aggressive and more resistant towards (immuno)therapies requires further study,

## Conclusion

In conclusion, we have demonstrated that several immune cell subsets can infiltrate pediatric CNS-PNETs, although the activation status of the cytotoxic cells (granzyme B) is variable. Pediatric CNS-PNETs might evade immune recognition by downregulating cell surface MHC-I and CD1d expression. We show that brain tumors can acquire expression of SERPINB1, SERPINB4, and SERPINB9 as a potential mechanism to resist granzyme-induced cytotoxicity. Although the mechanistic insights remain to be elucidated. This study hints to the putative implications of serpin expression for the success rate of immunotherapy in CNS-PNET patients. To predict clinical outcome and response to immunotherapy, further study is required in larger pediatric CNS-PNET cohorts as wells as in the currently running clinical trial of pediatric medulloblastomas/CNS-PNET immunotherapy, in which dendritic cell vaccination and adoptive cellular immunotherapy with cytotoxic lymphocytes is employed [Vaccine Immunotherapy for Recurrent Medulloblastoma and Primitive Neuroectodermal Tumor (Re-MATCH), University of Florida, NCT01326104].
